# COVID-19: the great unequaliser

**DOI:** 10.1177/0141076820925434

**Published:** 2020-06-10

**Authors:** Delan Devakumar, Sunil S Bhopal, Geordan Shannon

**Affiliations:** 1 Institute for Global Health, UCL, London WC1N 1EH, UK; 2 Population Health Sciences Institute, Newcastle University, Newcastle upon Tyne NE2 4BN, UK; 3 Northumbria Healthcare NHS Foundation Trust, Tyne and Wear NE29 8NH, UK

COVID-19 infection can lead to devastating consequences for individuals, families and wider society. But the impact on individuals is not equal. In an age of populist and divisive movements around the world, the outbreak has been racialised, hitting minority and marginalised communities the hardest.^1^

From the first known cases in Wuhan, China in December 2019, the infection was rapidly identified in nearby countries and then in Europe and North America. This spread was coupled with individual and more formalised acts of racism towards Chinese and East Asian people. This was both within China, with restaurants in Hong Kong barring people from mainland China for example, and in other countries, including attacks on Asian Americans.^2^ The virus has since spread all over the world but it is still described by many, including President Donald Trump, as the Chinese virus.^3^ This has historical roots: for example, the 1918 flu pandemic was commonly described as the ‘Spanish Flu’, despite there being no clear link to it originating in Spain. In the United States, the 1882 Chinese Exclusion Act was a racially driven United States policy barring immigration of Chinese nationals and blaming of Chinese migrants for disease outbreaks. Brazil’s Minister of Education, Abraham Weintraub, even said that COVID-19 is part of China’s plan for global domination.^4^

It has been said that COVID-19 is the great equaliser. Yes, celebrities and even the Prime Minister of the United Kingdom, Boris Johnson, can be infected and become severely unwell. But like all illnesses, how society chooses to collectively manage COVID-19 does, in fact, discriminate. While consideration of biological/physiological risk factors – including co-morbid illnesses – are rightly considered, the socially constructed impacts, based on ethnicity and migratory status, receive much less attention. The risk of contracting COVID-19, the severity of the illness and the risk of poor health related to the policies and actions responding to the pandemic are all increased in Black, Asian and minority ethnic and migrant groups. These amount to a form of structural violence, placing Black, Asian and minority ethnic people and migrants at greater risk.

First, Black, Asian and minority ethnic and migrant groups have a greater risk of contracting COVID-19 infection, as they are more likely to live in poor and overcrowded housing and do precarious forms of work or work in the gig economy. For many, stopping work is not just an inconvenience, it is impossible. This increases the chance of getting an infection and then it can more easily spread to family members in crowded and high-density dwellings. This is not to mention the 44% of the NHS medical workforce of Black, Asian and minority ethnic backgrounds, who are on the frontline treating patients with limited protection.^5^

Second, Black, Asian and minority ethnic groups are more likely to get a severe form of infection. The evidence to date shows that having a non-communicable disease increases the risk of hospitalisation from COVID-19.^6^ These diseases, such as diabetes or cardiovascular disease, tend to be more common in Black, Asian and minority ethnic groups. Data from the Centers for Disease Control and Prevention in the USA show that 33% of hospitalised patients were Black, compared to 18% in the population.^7^ Another important issue is that many migrant groups, especially those without documents, are less likely to seek help, or may seek help later, with more advanced disease.^8^ The introduction of the UK Government’s ‘hostile environment’ policy, including barriers to accessing the health service, such as upfront charging and the sharing of data with the Home Office, has led to migrants avoiding healthcare.^9^ Infectious diseases are exempt from hospital charges but the rules are complicated and many people fear going to hospital in the first place.

Third, it is likely that the UK, and the world more generally, will enter one of the deepest recessions in a lifetime. This is in addition to the financial crisis of 2008 and subsequent decade of ‘austerity’ which has eroded national health and social safety nets, as well as the anticipated years of uncertainty and trade negotiations related to Brexit. The poorest, with insecure employment, and most vulnerable in terms of health are then at risk for other stress-mediated health problems, especially mental health issues, that increase in times of recession.

In the longer term, we also need to be careful to ensure that current emergency policies are maintained only as far as they are required to combat COVID-19. While technology, for example contact tracing apps for surveillance, may prove to be useful, it can also easily infringe on human rights afterwards, with minority and marginalised groups being affected disproportionately.^10^

Infectious diseases have long been associated with ‘othering’ and minority groups are often blamed. Economic hardship is a fertile ground for populist movements to thrive and sadly, many world leaders have used the COVID-19 outbreak, mixing public health actions with divisive policies to further their own agendas. Minority and marginalised groups bear the brunt of this. Migrants are unfairly targeted as bringing infections into a country, when it is mostly business and frequent flying travellers and tourists. Take Victor Orban^11^ or Matteo Salvini,^12^ for example, who have linked COVID infections to illegal migrants coming into Hungary or Italy. Or Donald Trump’s reassertion that a wall with Mexico might help.^3^ In spite of mass suffering and death, why miss an opportunity to drive home your message? But policies that foster fear and division do not help anyone.

To successfully combat a pandemic, health protection measures rely on well-prepared and well-functioning health services that treat and support everyone, ensuring those most at risk are protected. Global health responses are not, however, limited to health service provision, but must be active in ensuring safe, inclusive and just social and structural conditions for everyone. Public health principles based around equity should be firmly at the core of the world’s response.

## Declarations

### Guarantor

DD
